# An integrative literature review on intimate partner violence against women in South Korea

**DOI:** 10.4069/kjwhn.2020.11.15

**Published:** 2020-12-14

**Authors:** Hye Young Min, Jung Min Lee, Yoonjung Kim

**Affiliations:** 1 Division of Nursing Science, Graduate School, Ewha Womans University, Seoul, Korea; 2 School of Nursing, University of North Carolina at Greensboro, USA

**Keywords:** Domestic violence, Intimate partner violence, Review, Women

## Abstract

**Purpose:**

The purpose of this study was to analyze and synthesize the literature on intimate partner violence (IPV) against women in South Korea.

**Methods:**

Whittemore and Knafl’s integrative review method was used. Studies in English and Korean were searched in seven electronic databases using the following combination of terms: “Korea,” “females or women or girls,” “intimate partner violence or domestic violence or domestic abuse.”

**Results:**

Twenty-five studies were ultimately selected, all of which met the quality appraisal criteria with a grade of medium or higher, using Gough’s weight of evidence. IPV was divided into marital violence and dating violence. Factors related to IPV were classified into intrapersonal, interpersonal, and social factors, and these three factors were linked together. Intrapersonal factors included general characteristics, perceptions, attitudes, psychological factors, and violent experiences. Interpersonal factors involved relationships with parents and partners. Finally, social factors and attributes were integrated into social support and influences on life.

**Conclusion:**

In order to minimize and prevent harm to women from IPV when caring for women who experienced IPV, multiple factors should be considered. Specifically, general and psychological characteristics, perceptions and attitudes toward IPV, relationships with families and partners, and available social support systems and resources should be considered. Moreover, these findings will be helpful for assessing women or providing interventions for victims of violence. Finally, more diverse IPV studies should be conducted by nurses in the future.

## Introduction

Intimate partner violence (IPV), which includes controlling behavior, is one of the most common forms of violence against women [1]. In the study of García-Moreno et al. [2], IPV was defined as “any behavior within an intimate relationship that causes physical, psychological, or sexual harm to those in the relationship”. In recent years, IPV has become an important public health issue. The incidence of violence against women, including sexual assault, is high worldwide [3], and despite ongoing movements and interest in fighting against violence - women, its incidence is not decreasing. According to a World Health Organization (WHO) report, 59% of women aged between 15 and 49 years worldwide have experienced forced sexual or physical abuse by their partners at least once [3]. A 2018 report in Korea also reported that at least 824 women had been killed, and at least 602 women had been placed at risk of death, in the past 9 years by IPV [4]. This finding indicates that IPV is also a problem that cannot be overlooked in Korea.

Violence, caused by a power imbalance in close relationships, has recently been acknowledged as a serious social problem that needs to be addressed [5]. The power imbalance within traditional gender norms and close relationships directly or indirectly increases the vulnerability to violence within close relationships, causing the victim to be receptive to the other party’s violent behavior [6]. Moreover, traditional gender norms and power disparities increase society’s tendency to accept traditional gender roles and prevent women from flatly rejecting male proposals for sex. This increases the vulnerability of women to become sexual victims and exacerbates their inability to refuse unprotected sex [6,7]. Previous studies [2,8] have shown that women with more stringent traditional gender norms have lower sexual self-assertion and self-efficacy within relationships, leading to an increase in the likelihood of experiencing sexual violence from their partners. These findings show that people who have no power in sexual relationships and who have a low level of sexual self-assertion are vulnerable to unwanted physical and mental harm, and are less capable of making actions or decisions related to sexual activities on their own. Women with these characteristics are more vulnerable to IPV, and efforts are needed to reduce its harm.

Sexual violence, in particular, is often caused by unilateral coercion during dating, without consent from the partner. IPV is often committed by dating partners [7], and the incidence of sex crimes (e.g., sexual violence, dating violence; wife-beating) continues to grow in Korea [9]. The percentage of people who reported dating violence reported in South Korea increased from 16.2% in 2016 to 19.9% in 2017 [10]. In addition, most perpetrators were male, and it was found that people in their 20s experienced more dating violence than in other age groups [10]. Another Korean study [11] also reported similar results: about 97% of victims of sexual violence were women. Of these, 64.4% were adult women, and 55.7% were in their 20s.

The physical or mental harm of violence against women is severe, both in the short and long term [12]. Victims of violence need immediate and long-term health care and psychological treatment. Violent experiences such as sexual assault, physical abuse, and stalking cause physical harm, which may include sexually transmitted diseases, as well as mental disorders such as post-traumatic disorder, depression, and insomnia [12]. In addition, because IPV occurs regularly and repeatedly due to the nature of the relationship between the victim and the perpetrator, women exposed to harm experience difficulties in forming supportive social relationships, have poor subjective health, and face limitations in daily activities [3,13]. Furthermore, violence reduces women’s ability to work and opportunities to care for their families and contribute to society. Children who witness violence at home are also more likely to have behavioral and emotional problems [14].

There is a lack of systematic reviews on IPV experienced by Korean women, as well as a lack of research in Korea that distinguishes gender and power on IPV against women. However, since IPV has recently been recognized to be caused by power imbalances within close relationships, integrated consideration of existing research on IPV is essential. Therefore, this study sought to analyze and evaluate studies of IPV against Korean women, with the aim of identifying the characteristics and attributes of prior studies related to IPV against Korean women.

## Methods

Ethics statement: This study is a literature review of previously published studies and was therefore exempt from institutional review board approval.

### Research design

This study is an integrative literature review that comprehensively analyzed research related to IPV against Korean women.

### Research procedure

In accordance with the integrative review guidelines of Whittemore and Knafl [15], the research questions were clarified; the literature was searched and selected; data evaluation, data analysis, and semantic analysis of selected papers were conducted; and five stages of integrated data extraction were carried out. In addition, a quality assessment of the selected studies was conducted using Gough’s weight of evidence (WOE) [16].

### Clarifying the research question

To clarify the research problems and objectives, the researchers described the research question and objectives at the beginning of the study. The main research question of this study was, ‘What does the nature of the studies related to IPV against Korean women show?’

### Search and selection of literature

The researchers wanted to find meaningful and appropriate materials that fit the research problems and objectives, and recorded the process in detail to increase the accuracy of the literature search and the reliability of the research. Through meetings, the three researchers prepared the final analysis based on the following selection criteria. The criteria for selecting the papers included in the literature review were (1) studies of Korean women, (2) studies that included the keyword “intimate partner violence,” (3) research papers published in Korean and international journals after peer review, and (4) studies in English or Korean. The criteria for exclusion from the literature review were (1) studies in which the study design was arbitration to see the effects of a literature review, tool development, and programs; (2) studies including males (because a separate analysis was not possible); and (3) government reports, dissertations, letters to editors, or papers published in academic conferences.

The first search and analysis of the literature was conducted from November 16 to December 16, 2019, and a second search was conducted from October 12 to October 21, 2020. There were no restrictions on the year of publication of the searched papers. The following three databases (DBs) were used: the Korean Studies Information Service System (KISS), the Research Information Sharing Service (RISS), and the National Digital Science Library (NDSL) in Korea; and the keywords used for the search were a combination of “women,” “IPV,” “intimate partner violence,” “close relationship violence,” and “close partner violence.” For international DBs, four search engines were used: Cumulative Index to Nursing and Allied Health Literature (CINAHL), PubMed, PsycINFO, and Scopus and the keywords were a combination of “Korea*,” “females or women or girls,” and “domestic violence or domestic abuse or intimate partner violence” (Supplementary Table 1).

**Figure. 1.**
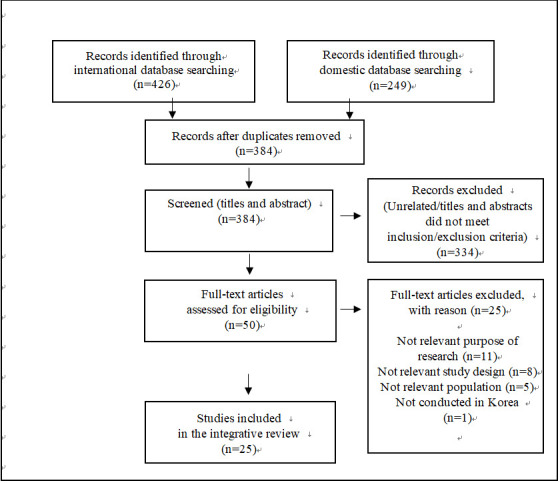
Flow diagram of study selection.

Using the combined search terms, a total of 675 studies were identified, including 249 studies from Korean DBs and 426 studies from foreign DBs. From this pool, the titles and the abstracts were reviewed based on the selection criteria. In total, 334 studies and 291 duplicates were excluded. After carefully examining the full text of the remaining 50 studies and further applying exclusion criteria, we finally selected 25 studies [17-41] (Figure 1).

### Evaluation of data

To evaluate the quality of the selected literature, Gough’s WOE [16] was used, and researchers assessed whether the research problems, research purposes, methods, selection of subjects, grounds, and results were described in the selected literature. The first item (WOE a) of the weighted value was evaluated with a focus on the context and evidence of the study by assessing the consistency and integration of the evidence presented in the study in relation to the research problem. The second item (WOE b) was to determine whether the form of evidence presented in the study in relation to the research question was consistent with the study question and purpose, thereby evaluating whether the study was properly designed for the study question and purpose. The third item (WOE c) evaluated whether the research methods or research analyses were appropriate for the research problems, and the studies were evaluated with items such as selection of subjects, data collection and data analysis, and ethical aspects. Finally, the fourth item (WOE d) was evaluated comprehensively based on the three preceding items. A study was classified as “medium” or “high” if two or more of the three items evaluated earlier were “high” or “medium.” However, in Gough’s study [16], there was no clear standard for ratings based on the evaluation scores, so in this study, we referred to the study of Haßler et al. [42], which clearly presented evaluation criteria using Gough’s WOE.

### Analyzing data and interpreting meaning

Analyzing data and interpreting meaning are steps to analyze the finally selected research through quality evaluation and to synthesize its meaning. The researchers reviewed all the data independently, then organized and analyzed them individually. When the researchers compiled the selected papers, they extracted the year of publication, study design, participants, purpose of research and research questions, variables, and results of research, and also discussed what other selected studies found on IPV among Korean women. Subsequently, each of the summaries was compared and reviewed through regular meetings, and six offline and online meetings were conducted to coordinate opinions and to reach agreement on discrepancies in the data. In addition, the decision-making and progress processes from the beginning to the end of the study were described in detail so that other researchers could clearly understand these aspects of the study and reach a reasonable level of agreement with the opinions that emerged through the analysis.

### Integration of data

The final step of the integrated consideration of study findings was to present how the attributes identified through the researchers’ consensus were derived from the data. The analysis was conducted using the reference matrix to derive the properties of the research, and the final properties were confirmed by checking whether the derived properties were closely linked to the main data.

## Results

Upon quality assessment, all 25 studies on IPV against Korean women analyzed in this study used data of medium or higher (Table 1). The characteristics of the analyzed studies were as follows (Table 2); fifteen studies (60.0%) were conducted among married women and 10 studies (40.0%) were conducted among single women. Quantitative studies were the most common (60.0%), of which 12 (48.0%) were descriptive studies. There were nine qualitative studies (36.0%) and one mixed-methods study (4.0%). Four studies on intimate relationship violence against women were published before 2011, 10 were published between 2011 and 2015, and 11 were published from 2016 to the present, indicating that studies related to women’s violence have been steadily published. The main variables of the reviewed papers related to marital violence (32.0%), dating violence (40.0%), and victims’ beliefs, attitudes, and influencing factors for each type of abuse (28.0%). Most studies were conducted in fields in the social sciences such as social welfare (n=7), psychology (n=5), and law and criminology (n=5), followed by nursing (n=5) and medicine (n=1). Although there were relatively few studies in the health field, the four studies published within the last 2 years were from nursing, suggesting an increasing interest in the study of IPV in nursing research.

### Analysis of data for selected papers

In this study, 25 studies on IPV were analyzed according to the study questions (Table 3). The 12 descriptive studies presented research on experiences of IPV [17-19], influencing factors [20-25], the legal and support system [26,27], and women who relied on the perpetrators of IPV [28]. The nine qualitative studies explored experiences of IPV [29-32], women’s experience of building their identity after IPV [33], experiences of seeking help to overcome IPV [34], and experiences after IPV ended [17,35]. Quantitative studies investigated the level of harm caused by IPV [36], predictors of risk [37], the relationship of IPV with depression [38], and gender awareness and self-assertion [39]. Among the major variables used in IPV studies were demographic and sociological characteristics related to marital status and household income [22,28,38], stereotypes such as dating violence beliefs or tolerance [17,24,25,36,39], and sexual self-assertion related to perceptions of violence against women [23,25,39]. In addition, studies on coping [29,31,32,34] and the coping process [31,32,35] were conducted in studies dealing with post-IPV experiences.

Women’s experiences of spousal violence were closely related to their experiences of parental verbal violence in childhood [18] and subsequently were associated with women’s depression, stress, and diminished self-esteem. The most commonly investigated risk factor was the experience of parental violence against the child, and if violence was experienced as a child, this was likely to become transposed to a violent relationship in adulthood [33,38]. Conversely, a study that evaluated the causal relationship between child abuse victims and adult dating violence offenders [21] found that experiencing child abuse by parents did not have a statistically significant impact on subsequent physical dating violence and youth harm. Those who experienced parental violence or watched their mothers be abused since childhood [30,33] or grew up in patriarchal home environments [22,28] developed stereotypes about gender roles and showed low sexual self-assertion as women [17,37]. Experiences of parental violence before adulthood affect interpersonal relationships due to depression and stress, and increase the risk of intimate violence after adulthood. The results of analyzing the selected studies suggest that steps must be taken to educate parents on how to correct their children’s behavior without physical punishment or verbal violence, and that mediation programs to prevent marital violence in the community should be implemented more actively.

Some of the studies [24,25,29,32,41] regarding dating violence showed that gender stereotypes and sexual self-assertion affected the acceptance of violence in close relationships [24,25]. Among victimized women exposed to parental violence in childhood, a learned sense of helplessness about violence appeared [20], the severity of abuse was not recognized in close relationships [28], and tolerance for violence was confirmed to be different [25,39]. In addition, women who experienced parental violence due to exposure to patriarchal attitudes in childhood were found to be at risk of becoming perpetrators and victims of violence in the future [17,22,24,28,31]. Perceptions of violence in intimate relationships varied among women who were victims depending on their knowledge about sexual violence [25] and implicit gender stereotyping [17,24,39], which has been shown to affect woman’s beliefs in and attitudes toward violence [36]. Adult attachment, self-understanding, self-impact factors [18,23], and sexual self-assertion [25,39] were found to affect post-violence attitudes in close relationships.

A secondary study of IPV [27,36] assessed the level of harm to victimized women and their beliefs, attitudes, and depression about violence. Tolerances or attitudes toward violence against women in Korea were related to educational level [22,24,27,38], previous experiences of parental violence [17,19,21], and adult attachment [23,30,33]. An analysis of the general characteristics of women who had experienced violence [23,24,27] showed that the awareness, severity and tolerance of violence were associated with unstable employment status, low income level, and low education level. Among women affected by IPV, with high levels of education and easy access to social support services, attempts have been made to overcome experiences of violence by seeking help and using support systems in the local community [20,25,26]. In addition, studies conducted among women revealed that higher levels of sexual assertiveness were associated with less tolerance of dating violence and lower gender role stereotypes [25,39].

In this review, studies on the coping and overcoming of IPV among Korean women were examined, including changes after the relationship with the perpetrator ends [29,35], and requests for help in the process of overcoming IPV [34]. Besides, the results of this review, IPV should be assessed in the local community as a way to overcome violence [18,21,28], and in order to prevent violence, education on gender stereotypes [22,24,26] and violence [17-19,28,39] is needed. It was also found that women who already have experienced IPV need education for increased sensitivity to violence, as well as counseling and psychotherapy to prevent subsequent trauma, such as depression and distress [20,29,36,38,41].

### Interpretation of meaning

The factors that influenced women before the occurrence of IPV included previous experiences of violence, growth environment, beliefs and attitudes, gender stereotypes, gender stereotypes, and sexual self-assertion. The types of IPV were broadly divided into marital violence and dating violence, which in turn could be classified as physical, mental, and sexual violence. However, types of violence in close relationships occurred in association with each other rather than independently. After the occurrence of IPV, the impacts of IPV on women included depression, stress, and anxiety. The victim’s attitude toward overcoming violence and ways of coping with it affect the likelihood of becoming a victim of IPV again.

### Integration of data

In this study, IPV-related factors were grouped into intrapersonal factors, interpersonal factors, and social factors, and these three factors were linked together (Table 4). First, intrapersonal factors included women’s general characteristics and psychological factors, awareness and attitudes toward IPV, and past experiences of IPV. Specifically, psychological factors included depression, stress, self-understanding, awareness of IPV, knowledge and beliefs, sexual self-assertion, and gender role stereotypes, including awareness of IPV and attitudes toward IPV. In addition, past experiences of IPV included parental violence and child abuse. In addition, the findings showed that personal experiences on parental violence or dating violence and gender norms influenced individuals’ ability to ask for help or manage violence in close relationships. Second, interpersonal factors referred to the relationships between individuals, in addition to the characteristics of the individual. These relationships can be divided into parents and partners, including spouses. Women who suffered violence in intimate relationships were affected by witnessing patriarchal attitudes, parental violence, and mother-targeted violence in childhood. Their relationships with partners affected their experiences of marital violence, the severity of physical violence, the risk of spousal assault, the consequences of violence, their active response to spousal violence, and their tolerance of dating violence. The researchers identified the formation of intimate violence in relationships with new partners and the risk that past victims may become perpetrators. Third, social factors included the impact of social support, such as the degree of counseling assistance, the relationship with the counselor, and the social support system after IPV. Furthermore, analyses of the influence of IPV on life before or after the harm showed that the victim-survivors of IPV experience numerous negative consequences even after their abusive relationships end [32,35].

## Discussion

This study found that research on IPV has been consistently published since 2010. The incidence of IPV in Korea has decreased compared to the past, but it still has a higher profile than that of other countries [43]. This warrants continuing attention. Most studies on IPV were conducted in the social sciences, and only five of the 25 studies were in nursing, suggesting the need for more intensive nursing research on IPV. The WHO recognizes IPV as a public health issue and emphasizes the need for an appropriate understanding among health care providers, including nurses [12]. Nurses are more likely to encounter IPV victims [44] and spend the longest time with patients compared to other health care providers [45]. Therefore, nurses must be sufficiently prepared to face and treat women who report instances of IPV. In a study of nurses’ roles and preparation for IPV that was conducted in India [46], the results showed positive correlations among the level of educational preparedness, self-efficacy, and attitudes toward caring for women affected by IPV. Another study confirmed that simulation education for IPV was significantly helpful for preparing nursing students to care for women affected by IPV [47]. Thus, conducting more diverse studies of IPV for nursing education in Korea will be beneficial.

The factors related to IPV can be classified into intrapersonal, interpersonal, and social factors. Intrapersonal factors (e.g., depression, stress, and experiences of violence in the past) were identified as risk factors of IPV in a prior study [48], and therefore the psychological and social aspects of IPV must be assessed when caring for victims. In addition, for adult women who have not experienced IPV or have not suffered harm, psychological and social aspects should be assessed to prevent and minimize harm, and nurses must identify and link resources that can be utilized within the community in advance if necessary. As experiences of parental violence and child abuse while growing up appears to affect adulthood, education about and screening for pre-adult IPV is also necessary [48]. So far, however, preventive education for IPV has been insufficient. Since violence prevention education mainly deals with sexual violence for school-age children, adolescents, and young adults [49,50], education should therefore include more a comprehensive and wider range of knowledge about IPV.

In addition, nurses should be prepared to consider combinations of intrapersonal factors. In other words, for nurses to deal with IPV, a wide range of understanding and knowledge is needed through education. If nurses are prepared to provide nursing care on the basis of a sufficient knowledge of IPV, they will have a positive attitude and self-efficacy when caring for women [46]. Therefore, if nurses understand the characteristics of women vulnerable to violence in intimate relationships (e.g., low education, being young and married) [43], and reflect that understanding in their care, they will be able to have a positive impact on the women as well as on themselves.

Second, interpersonal factors are those related to relationships with others in addition to individual characteristics. As the notion of IPV [1] involves all forms of violence in close relationships, IPV can be broadly divided into relationships with parents and partners, including previous parental violence, as well as dating violence. Therefore, in order to provide an assessment and mediation of IPV, it is necessary to implement parental education [18] as well as marital and partner education as precautionary interventions. Partner training should be aimed toward teenagers and college students who are potential partners in the future. The training should also include both male and female teenagers and college students, rather than targeting only men, considering the diverse gender identities of women.

Lastly, for social factors, women affected by IPV are more likely to comply with the perpetrator’s demands than to break away from the violent relationship [29]. Thus, there is a need for interventions addressing social factors that enable women to overcome subsequent trauma. For example, attitudes toward IPV may be changed through counseling, which may help women to cope more effectively with the effects of IPV. For this purpose, at the community level, women exposed to danger should be assessed and protected from harm [18,28]. In addition, social and policy interventions are necessary. In Korea, the Framework Act on the Prevention of Violence against Women was enacted in 2018 to protect women from various forms of violence, including violence in close relationships. The Act was established following a nationwide descriptive survey, aimed to protect women who have been exposed to violence and to protect and support the victims. In addition, there are currently hotlines in Korea dedicated to women that provide counseling on violence against women, linkage and supportive resources for victims, training in preventing violence, and activities to increase awareness of IPV through telephone support [4]. Therefore, nurses need to be familiar with the laws enacted and related available resources to be able to make full use of social and policy aspects, as well as personal preparation.

This study has some limitations. The search was limited to research papers on IPV affecting women, in order to comprehensively examine the experience of IPV against Korean women. In addition, although the researchers made efforts to conduct a systematic and comprehensive search and selection, it is possible that some relevant papers were missed in the search phase or in the title and abstract review phase. Finally, as this study focused on research related to IPV against Korean women in Korea, there is a limit to generalizing the results to IPV against migrant women living in Korea or Korean women living abroad.

This study examined 25 studies on IPV against women in Korea and, through the meaning analysis of IPV, identified important factors that influenced IPV before its occurrence, types of violence upon occurrence, and the process of overcoming IPV after its occurrence. It was also confirmed that IPV affects women in terms of intrapersonal, interpersonal, and social factors, which are closely interlinked. The results of this study are significant in that they include matters that nurses may consider in preventing IPV and caring for victimized women. Specifically, general and psychological characteristics, perceptions and attitudes toward IPV, relationships with families and partners, and available social support systems and resources should be considered. The results of this study can be used for developing educational content in the required curriculum for teenagers and college students, by including content on intrapersonal, interpersonal, and social factors of IPV. The findings can also guide IPV assessment and the provision of arbitration for victims.

Since nursing research on IPV is limited, albeit having increased in the past 2 years, more IPV research by nurses is needed. This includes studies that focus on nurses, such as descriptive studies on nurses’ knowledge and perceptions of IPV, as well as nursing education for IPV care readiness.

## Figures and Tables

**Table 1.** Quality appraisal of reviewed articles (N=25)

First author [Ref No.]	Year	Gough’s WOE (WOE a, WOE b, WOE c, WOE d)
Ko [35]	2020	H, H, H, H
Park [34]	2020	H, H, H, H
Kwon [29]	2019	H, H, H, H
Kwak [23]	2018	H, H, M, H
You [31]	2018	H, H, H, H
Hong [20]	2017	H, H, M, H
Kim [19]	2017	H, H, H, H
Woo [32]	2017	H, H, H, H
Park [41]	2017	H, H, H, H
Shon [39]	2016	H, H, M, H
Park [24]	2016	H, H, M, H
Hong [26]	2015	H, H, M, H
Bae [33]	2014	H, H, H, H
Choi [17]	2014	H, H, M, H
Park [30]	2014	H, H, M, H
Jennings [21]	2014	H, H, H, H
Kim [38]	2013	H, H, M, H
Kim [37]	2013	H, H, M, H
Han [36]	2012	H, H, M, H
Park [25]	2012	H, H, M, H
Hong [40]	2011	H, H, M, H
Kim [27]	2010	H, H, H, H
Kim [28]	2010	H, H, M, H
Kim [18]	2009	H, H, M, H
Kim [22]	2009	H, H, M, H

Ref No.: reference number; WOE: weight of evidence.

H: high; M: medium; WOE a: methodological trustworthiness; WOE b: methodological relevance; WOE c: topic relevance; WOE d: overall score.

**Table 2.** General characteristics of the included studies (N=25)

Characteristic	Categories	n (%)	Reference number
Participants	Single women	15 (60.0)	[17], [19], [20], [21], [23], [25], [27], [29], [31], [32], [34-36], [39], [41]
	Married women	10 (40.0)	[18], [22], [24], [26], [28], [30], [32], [33], [38], [40]
Research design	Quantitative descriptive study	12 (48.0)	[17-28]
	Qualitative study	9 (36.0)	[29-35], [40], [41]
	Secondary data analysis	3 (12.0)	[36-38]
	Mixed-methods study	1 (4.0)	[39]
Publication year	Before 2011	4 (16.0)	#x0005b;^18^], [22], [27], [28]
	2011-2015	10 (40.0)	[17], [21], [25], [26], [30], [33], [37], [38], [40]
	2016 and beyond	11 (44.0)	[19], [20], [23], [24], [29], [31], [32], [34], [35], [39], [41]
Sample size	Less than 100	13 (52.0)	[17], [29], [20], [28-35], [40], [41]
	101-500	7 (28.0)	[23-27], [36], [37]
	501-1,000	2 (8.0)	[22], [39]
	>1,000	3 (12.0)	[18], [21], [38]
Main variables	Marital violence	8 (32.0)	[18], [20], [24], [26], [28], [30], [33], [40]
	Dating violence	10 (40.0)	[17], [21], [22], [29], [31], [32], [34], [35], [39], [41]
	Factors affecting violence	7 (28.0)	[19], [23], [25], [27], [36], [37], [38 ]
Study field	Social welfare	7 (28.0)	[18], [19], [24], [26], [30], [32], [38]
	Counseling	2 (8.0)	[31], [41]
	Psychology	5 (20.0)	[17], [20], [25], [32], [40]
	Law and criminology	5 (20.0)	[21], [22], [26], [28], [37]
	Nursing	5 (20.0)	[23], [29], [34-36]
	Medicine	1 (4.0)	[39]

**Table 3.** Major contents of the reviewed articles (N=25)

First author (year)	Design	Participant (sample size)	Purpose/research question	Main variables	Key findings
Ko (2020) [35]	Qualitative study	Single women (13)	To understand young female adults' experiences of building a new intimate relationship after ending their abusive relationship	• Exploring IPV experiences	• The victim-survivors of IPV experienced numerous negative consequences even after their abusive relationships ended.
				• Process of escaping IPV relationships	
				• Experiences after the IPV relationship had ended	• Care, safety planning, and emotional support for IPV victims are needed.
				• Experiences of new intimate relationships	
Park (2020) [34]	Qualitative study	Single women (14)	To understand South Korean female IPV victims' experiences in seeking help	• Experiences when seeking help according to selection of supporters	• Revealed the victims' experiences according to their choice of support and noted four factors that appear to influence their support selection, which were (a) recognition of the consequent harm after seeking help, (b) recognition of serious danger, (c) recognition of the probability of coping, and (d) recognition of the relationship.
				• Factors influencing supporter selection	
Kwon (2019) [29]	Qualitative study	Single women (14)	To explore the experiences of being coercively controlled in female victims who had experienced dating violence	• Experience of dating violence	• Starting the relationship by idealizing it (period of potential control)
				• Coping with dating violence	• Facing with visible coercive control (period of coercive control)
				• Changes after the end of the relationship	• Escaping from the unending trap (period of post-control)
				• Life impact of violent experiences	
Kwak (2018) [23]	Quantitative descriptive study	Single women (137)	To investigate the effects of adult attachment, responsibility attribution, and self-esteem of adult women on psychological aspect in intimate relationships	• Adult attachment	• The older, more dating experiences, and higher sense of responsibility, the higher the harm of psychological violence.
				• Responsibility attribution	
				• Self-esteem	• The older, more dating experiences, and more adult attachment, the higher the harms of psychological violence.
				• Psychological violence	
You (2018) [31]	Qualitative study	Single women (17)	To explore how women affected by dating violence experience the psychology of increasing levels of physical and sexual violence from mental violence	• Beginning of a relationship	• Causal conditions: weird, painfully pleasant, embarrassed, and scared
				• First IPV experience	• Contextual conditions: experiencing acceptance of violence and unusual love
				• IPV coping	• Arbitral conditions: level of experience of violence, the level of violence response, and the level of relationship immersion affect
				• Relationship with parents	
				• What would have changed if I had realized that IPV had occurred?	
Hong (2017) [20]	Quantitative descriptive study	Single women (65)	To explore factors affecting the battered women's decision to return to the abusive relationship	• Learned helplessness	• The more learned helplessness and the more severe IPV, the less likely the battered women could escape from abusive relationships.
				• Social support	
				• Spousal assault risk	
Kim (2017) [19]	Quantitative	Single women (16)	To examine psychological difficulties and stress levels in abused and non-abused Korean women and analyze the relationship between psychological outcomes and stress level	• Psychological variables	• Women who experienced IPV had more life stress events and lower antioxidant levels than non-abused women.
				• Oxidative stress biomarkers	
					• Relationships between a women's physical health and life stress arising from IPV had significant implications.
Woo (2017) [32]	Qualitative study	Single women (7)	To understand the process and contextual factors of victims’ experiences of dating violence	• Experience of overcoming dating violence	• The central phenomenon experienced by women affected by dating violence was the being trapped in a violent situation.
					• Unstable parenting environment, such as parental abuse, and loving interpretation of the perpetrator's excessive care and controlling behavior
					• Analysis of the process of dating violence experience with trial steps, tightrope steps, recovery phases, and growth phases
Park (2017) [41]	Qualitative study	Single women (6)	To explore the experiences of being coercively controlled in dating violence victims	• Experience of being coercively controlled	• Idealizing the relationship (period of potential control)
					• Facing severer tyranny (period of coercive control)
					• Escaping from the unending trap (period of post-control)
Shon (2016) [39]	Mixed method Single women study (548)		To verify the impact of the continuation of dating violence on girls as a cognitive process	• Gender role stereotypes	• Internal reasons included low self-esteem, fear, fear, anxiety, excessive altruism, and a desire not to be abandoned.
				• Allowing dating violence	
				• Sexual self-assertion	• A significant correlation was found between gender stereotypes of college students, tolerance for dating violence, and sexual self-assertion.
				• Qualitative: reasons for sexual assertions and how to asking help	
Park (2016) [24]	Quantitative descriptive study	Married women (150)	To determine the levels of violence perceived by female victims of IPV and to explore factors other than gender stereotypes in multilateral aspects	• Type of abuse (physical, emotional, and sexual abuse)	• IPV usually exhibited two or more forms.
				• Gender stereotypes	• The higher the violence experience, the higher the gender stereotypes.
					• The lower the level of education, the more experience they had of IPV.
Hong (2015) [26]	Quantitative descriptive study	Married women (222)	To explore the reasons why battered married women do not ask for help from legal and institutional systems	• Active reactions to spousal violence	•A permissive attitude toward violence in Korea disrupted victims' questions for outside help
Bae (2014) [33]	Qualitative study	Married women (1)	To understand how identity is constructed for a woman who has experienced multiple violence	• Experiences of marital violence and identity of IPV	•The woman reconstructed her identity from “a bad woman” who deserved the violence to “a good mother,” not referring to ideology of mothering but “goodness” as value for her to reflect upon.
Choi (2014) [17]	Quantitative descriptive study	Single women (52)	To explore parental violence victim experiences and parental violence witnessed	• Parental violence experiences	•Parental violence experiences are noted for implicit gender stereotypes and dating violence experiences.
				• Dating violence experience	• Those who experienced parental violence had high levels of stereotypes.
				• Implicit gender stereotypes	• Experience of parental violence before adulthood affects interpersonal relationships in adulthood.
					• Children exposed to parental violence are at risk of exposure to adult dating violence.
Park (2014) [30]	Qualitative study	Married women (12)	To explore mothering experiences of battered women in the context of marital violence	• Experience of mothering	• Women who experienced violence had experiences that could be explained as “keep their lives going on by taking care of responsibilities and maintaining relationships with children.”
Jennings (2014) [21]	Quantitative descriptive study	Single women (1,252)	To estimate the effect of experiencing child physical abuse on dating violence among South Korean emerging adults	• Physical dating violence perpetration and victimization	• Child maltreatment is linked to later dating violence through mechanisms of social learning.
				• Physical child abuse	
Kim (2013) [38]	Secondary data analysis	Married women (3,153)	To examine the relationships between IPV and depression	• Level of physical violence	• Experiencing IPV influenced woman's level of depression in terms of its overall level and rate of change.
				• Depression	
				• Social support	
				• Household income	
Kim (2013) [37]	Secondary data analysis	Married women (119)	To explore the risk factors for predicting IPV in the framework of feminism theories	• Type of abuse (physical, emotional, and	• Predictable risk factors varied according to the type of IPV
				sexual abuse)	• Socioeconomic status incompatibility was related to IPV.
Han (2012) [36]	Secondary data analysis	Single women (172)	To explore IPV victim's harm level, IPV beliefs and attitudes toward IPV	• Female abuse assessment	• Beliefs and attitudes regarding IPV had a significant relationship with harm, depression, and IPV.
				• Beliefs and attitudes toward IPV	
				• Depression	
Park (2012) [25]	Quantitative descriptive study	Single women (240)	To investigate the factors affecting sexual assertiveness of female university students in order to prevent university students from having sexual problems	• Sexual harm and abuse	• Sexual assertiveness of female university students was correlated with the common idea of sexual violence, sexual experience (negative), and tolerance toward violence on dating (positive).
				• Common ideas of sexual violence	• Sexual assertiveness in university students influenced sexual intercourse experience and common ideas of sexual violence.
				• Tolerance toward violence in dating	
				• Sexual assertiveness	
Hong (2011) [40]	Qualitative study	Married women (6)	To explore the experience of women who left their abusive husbands	• Experience of leaving husband-to-wife violence	• Main themes were “keeping severing the violence with strength,” “self-exploring/accepting/understanding,” “strength gained/lost by outer factors,” “reconstruction of intimate relationship,” and “lingering problems and new hopes.”
Kim (2010) [27]	Quantitative descriptive study	Married women (124)	To examine the factors related to use of formal and informal resources by battered women	• Severity of physical violence	• The demographic characteristics, severity of physical violence, violence-related consequences, and partner child abuse were somewhat predictive of how battered women sought help.
				• Violence-related consequences partner and child abuse	
Kim (2010) [28]	Quantitative descriptive study	Married women (95)	How are abused South Korean women who resort to lethal violence against their abusers different from those who do not?	• Abuse	• Increased education and income levels may reduce women's tolerance for intimate partner abuse, as well as increasing the likelihood of seeking outside help when such abuse does occur.
				• Patriarchal attitude	• Patriarchal attitudes were negatively correlated with four variables (psychological abuse, education, physical abuse, and employment).
				• Marriage-related variables	
				• Socio-demographic measures	
Kim (2009) [18]	Quantitative descriptive study	Mamed women (1,079)	To examine the relationship between different types of marital violence, previous parental violence, and the mental health status of Korean women	• Self-esteem	• Both physical marital violence and previous parental verbal abuse had significant relationships with depression, stress, and aggression (i.e., mental health).
				• Depression	
				• Stress	
				• Aggression	
Kim (2009) [22]	Quantitative descriptive study	Married women (531)	To add knowledge about spousal homicide in non-Western cultures	• Severity of abuse	•The model for abuse history, patriarchal attitudes, and risk-taking preference were statistically significant, explaining 56.6%, 18.4%, and 4.1% of variance, respectively.
				• Strength of patriarchal attitudes	
				• Risk-taking preference	

IPV: intimate partner violence.

**Table 4.** Integrative description of intimate partner violence

Attribute	Categories	Reference number
Intrapersonal factor	General characteristics	[28], [30], [41]
	Perceptions of IPV	[19], [20], [25], [31], [36-38], [41]
	Attitudes toward IPV	[19], [22], [29], [31]
	Psychological factors	[18], [28], [37], [38]
	Violence experience	[19-21], [29-32], [34-36], [40]
Interpersonal factor	Relationships with parents	[23], [24], [30], [31], [41]
	Relationships with partner	[17], [24], [26-30], [33-35]
Social factor	Social support	[28], [33], [39]
	Influence on life	[29], [35], [39]

IPV: Intimate partner violence.

## Supplementary materials

Further details on supplementary materials are presented online (available at https://doi.org/10.4069/kjwhn.2020.11.15).

Supplementary Table 1. Search Strategy
