# Supplementary material for: An integrative literature review on intimate partner violence against women in South Korea
Source: Korean J Women Health Nurs. 2020 Dec 14;26(4):260–73. doi: 10.4069/kjwhn.2020.11.15 (PMC9328617; doi:10.4069/kjwhn.2020.11.15)
Supplement: Supplementary Table 1. — Search Strategy [file kjwhn-2020-11-15-suppl.pdf]

KJWHN

Supplementary Table 1. Search Strategy

| Database | | Search term | Result |
| --- | --- | --- | --- |
| RISS | 1. | intimate partner violence AND 여성, 자료유형: 국내학술논문 | 41 |
| | 2. | 친밀 관계 폭력 AND여성, 자료유형: 국내학술논문 | 56 |
| | 3. | 친밀한 관계 폭력 &여성, 자료유형: 국내학술논문 | 56 |
| | 4. | 친밀한 파트너 폭력 &여성, 자료유형: 국내학술논문 | 5 |
| | Total | | 158 |
| KISS | 1. | intimate partner violence AND 여성, 자료유형: 학술지 | 12 |
| | 2. | 친밀 관계 폭력 AND여성, 자료유형: 학술지 | 24 |
| | 3. | 친밀한 관계 폭력 AND여성, 자료유형: 학술지 | 13 |
| | 4. | 친밀한 파트너 폭력 AND 여성, 자료유형: 학술지 | 3 |
| | Total | | 52 |
| NDSL | 1. | intimate partner violence AND 여성, 검색범위: 국내논문, 자료유형: 저널 | 20 |
| | 2. | 친밀 관계 폭력 AND여성, 검색범위: 국내논문, 자료유형: 저널 | 9 |
| | 3. | 친밀한 관계 폭력 AND여성, 검색범위: 국내논문,자료유형: 저널 | 9 |
| | 4. | 친밀한 파트너 폭력 AND 여성, 검색범위: 국내논문, 자료유형: 저널 | 1 |
| | Total | | 39 |
| CINAHL | (females or women or girls) AND (intimate partner violence OR IPV OR domestic violence OR domestic abuse) AND "Korea*" | | 60 |
| | Limits – Full text; Language: English, Korean | | |
| Pubmed | (females or women or girls) AND (intimate partner violence OR IPV OR domestic violence OR domestic abuse) AND "Korea*" | | 50 |
| | Additional filters – Text availability: Full text & Free full text; Species: Humans; Languages: English, Korean; Sex: Female | | |
| PsycINFO | (females or women or girls) AND (intimate partner violence OR IPV OR domestic violence OR domestic abuse) AND "Korea*" | | 103 |
| | Limits – Peer reviewed; Language: English, Korean; Population: Female; | | |
| Scopus | (females or women or girls) AND (intimate partner violence OR IPV OR domestic violence OR domestic abuse) AND "Korea*" | | 213 |
| | Limits – Peer reviewed; Document type: Article; Country/territory: South Korea; Source type: Journal; Language: English, Korean; Population: Female; | | |

IPV, intimate partner violence.

https://doi.org/10.4069/kjwhn.2020.11.15
